# Functional Activity of Plasmid DNA after Entry into the Atmosphere of Earth Investigated by a New Biomarker Stability Assay for Ballistic Spaceflight Experiments

**DOI:** 10.1371/journal.pone.0112979

**Published:** 2014-11-26

**Authors:** Cora S. Thiel, Svantje Tauber, Andreas Schütte, Burkhard Schmitz, Harald Nuesse, Ralf Moeller, Oliver Ullrich

**Affiliations:** 1 Institute of Anatomy, Faculty of Medicine, University of Zurich, Zurich, Switzerland; 2 Department of Machine Design, Engineering Design and Product Development, Institute of Mechanical Engineering, Otto-von-Guericke-University Magdeburg, Magdeburg, Germany; 3 Airbus DS, Airbus-Allee 1, Bremen, Germany; 4 Institute of Medical Physics and Biophysics, University of Muenster, Münster, Germany; 5 German Aerospace Center (DLR e.V.), Institute of Aerospace Medicine, Radiation Biology Department, Research Group Astrobiology, Linder Hoehe, Cologne (Köln), Germany; 6 Zurich Center for Integrative Human Physiology (ZIHP), University of Zurich, Zurich, Switzerland; University of South Florida College of Medicine, United States of America

## Abstract

Sounding rockets represent an excellent platform for testing the influence of space conditions during the passage of Earth's atmosphere and re-entry on biological, physical and chemical experiments for astrobiological purposes. We designed a robust functionality biomarker assay to analyze the biological effects of suborbital spaceflights prevailing during ballistic rocket flights. During the TEXUS-49 rocket mission in March 2011, artificial plasmid DNA carrying a fluorescent marker (enhanced green fluorescent protein: EGFP) and an antibiotic resistance cassette (kanamycin/neomycin) was attached on different positions of rocket exterior; (i) circular every 90 degree on the outer surface concentrical of the payload, (ii) in the grooves of screw heads located in between the surface application sites, and (iii) on the surface of the bottom side of the payload. Temperature measurements showed two major peaks at 118 and 130°C during the 780 seconds lasting flight on the inside of the recovery module, while outer gas temperatures of more than 1000°C were estimated on the sample application locations. Directly after retrieval and return transport of the payload, the plasmid DNA samples were recovered. Subsequent analyses showed that DNA could be recovered from all application sites with a maximum of 53% in the grooves of the screw heads. We could further show that up to 35% of DNA retained its full biological function, i.e., mediating antibiotic resistance in bacteria and fluorescent marker expression in eukariotic cells. These experiments show that our plasmid DNA biomarker assay is suitable to characterize the environmental conditions affecting DNA during an atmospheric transit and the re-entry and constitute the first report of the stability of DNA during hypervelocity atmospheric transit indicating that sounding rocket flights can be used to model the high-speed atmospheric entry of organics-laden artificial meteorites.

## Introduction

### Search for biosignatures of life

In the field of astrobiology a variety of research questions are addressed including the existence of life beyond Earth as well as the impact of space and extraterrestrial environmental conditions on biomolecules and different types of organisms [1]–[6]. Many projects have been launched with the goal to unambiguously identify signs of life on the planets and small bodies of our solar system. Recently, the Mars Science laboratory (MSL) was launched in November 2011 [7]. The MSL has, besides the characterization of geological, geochemical, planetary and radiation processes, the goal to study organic carbon compounds, the chemical building blocks of life (C, H, O, N, P, S) as well as the existence of biosignatures, representing features of biological processes on Mars [8]. A wide variety of biomarkers is investigated, natural products of biological origin, used to identify traces of past or present life. Organic compounds are very powerful biomarkers, especially those whose structures show long- term preservation [8]. Biomarkers, usually made of complex organic compounds are e.g. macromolecules like proteins as well as nucleic acids like DNA and RNA which represent the essential basis in all living organisms on Earth. Another highly sophisticated experiment searching for biomolecular evidence of life is the planned: The Icebreaker life mission to Mars representing a search for the biomolecular evidence for life [9]. This mission will focus among others on the search for biomolecules indicating that there was or still is life on Mars, the search for organic molecules in the ground ice and survey the environmental conditions for elements and energy sources required by potential life [9].

It is conceivable that life exists independently from our planet even under the very hostile conditions prevailing on our neighbours like e.g. Mars, since already on Earth we are able to identify some extreme life forms which can survive physically and or geochemically harsh conditions, such as very high or low temperatures, intense radiation, pressure, vacuum, desiccation, salinity, and pH (reviewed in [10]). Many of these parameters also prevail in space and therefore the question is whether terrestrial organisms are able to survive a voyage through space.

### Experimental platforms for microbial survival under space conditions

Various experimental platforms were used so far to investigate this question (reviewed in [11]). These platforms were often located in low Earth orbit (LEO), e.g. in the early 1990s, the development of a facility, that was designed for short-term, up to two weeks, space exposure experiments for biological and material samples (BIOPAN), was initiated by the European Space Agency (ESA) [12]. This facility, which is conceived for multi-user purposes, was mounted on the external surface of the descent module of the Russian Foton satellite. Astrobiology, radiobiology, and material science experiments have been performed on BIOPAN analyzing the effects of solar UV radiation, space vacuum, extreme temperatures, and microgravity on biological samples [12]–[17]. Nowadays platforms with even longer exposure times exist like the multi-user facility, EXPOSE, which is located on the outer platforms of the International Space Station (ISS). Different test samples of microbial and eukaryotic origin were subjected to space vacuum for 1.5 years, solar electromagnetic radiation, cosmic radiation and simulated Martian surface conditions and after sample recovery and analysis survival of a fraction of the tested organisms could be shown [18]–[24].

A critical question is if microorganisms do not only survive the residence in space but if they would also be able to withstand the hostile conditions of entering a planets atmosphere when they are situated e.g. on a meteorite. Due to velocities of 10–20 km/s, the material is heated by friction and the surface melts. However, the atmosphere transit time is only in the range of a few seconds preventing that the heat is penetrating the rock more than a few centimeters. In fact temperatures of the interior material of some Martian meteorites stayed far below 100°C [25]. Many microorganisms can survive and still metabolize under these conditions, e.g. *Bacillus subtilis* spores can survive moist heat with temperatures of up to 100°C for 20 to 30 min whereas, in dry heat spores survival increases by a factor of 1000 [26]. Also, spores of *B. subtilis* strain WN511, embedded in rock samples, survived temporarily at 145°C during the re- entry phase of a ballistic flight with 1.3 km/s on a sounding rocket when not directly exposed to the harsh re-entry conditions directly at the leading edge [27]. However, in the two atmospheric entry simulation experiments STONE-5 and STONE-6, for which spores of *B. subtilis* and *Ulocladium atrum* and the cyanobacterium *Chroococcidiopsis* sp. were embedded in different rock samples mounted on the heat shield of the Foton-M2 capsule, no viable microorganism were recovered after the flight [28]–[30].

The observed microorganism die-off is among others due to damages on the DNA level. The high radiation in space leads to formation of thymine dimers as well as single and double strand breaks and activates the UV repair mechanisms inside the cell, which often can not cope with the extent of DNA damage (reviewed in [4], [24], [31]–[33]). Additionally, the extreme temperatures, especially the high temperatures during the entry into a planets atmosphere are detrimental for the complete organism if it is not well protected. Also on DNA level high temperatures show extreme effects. With increasing temperature the DNA double strand melts and depending on the sequence and nucleobase composition results at a specific melting temperature in two single strands [34]–[36]. With higher temperatures the DNA molecules start to degrade by heat-induced weakening of the phosphodiester bond and subsequent hydrolysis [37]–[38].

### Sounding Rockets as research platforms for astrobiological experiments

Experimental time on the ISS and Foton satellites is difficult to aquire because of rare flight opportunities, a highly selective and time-consuming assortment of projects requiring the highest security standards at the same time. A suitable alternative to these long-term missions is the execution of experiments on sounding rockets [39]. Whenever microgravity and space-time in the range of minutes is sufficient these platforms are the means of choice. Sounding rocket programs on different launch vehicles (e.g. Mini-TEXUS, TEXUS and MAXUS) offering microgravity times from 3 to 13 minutes with guaranteed quality of 10^−4^ g reaching normally up to 10^−6^ g. During these missions altitude ranges from 150 up to 800 km are reached which allow to expose samples not only to microgravity but also to space conditions including, vacuum, radiation and extreme temperatures when mounted on the outside of the payload [39]–[41]. This provides an excellent test platform for astrobiology research especially for the analysis and characterisation of biomarkers. Samples can be easliy applied to the outside of the payload, sensors monitoring space conditions could be attached in the close vicinity of the samples, very short late access and early retrieval times in the range of a few hours can be achieved, safety requirements are less rigorous compared to manned missions and experiment equipment is less costly and the payload is reusable. Another advantage is the high launch frequency with which experiments can be performed and validated. Within the scope of the European space programs 10 to 15 sounding rocket missions are launched every year representing a potential high throughput testplatform for astrobiology research especially in the field of biomarker characterization.

### Biomarker analysis

Since DNA plays an important role as a major biomarker for the search of extraterrestrial signatures of life, it is important to characterize and compare the influence of space conditions and conditions on Earth. If we are searching for biomarkers on meteorites that reached our planet from millions of years ago to recently [42]–[43] it is of high interest to know more about the stability and quality of such a molecule after being ejected from another planet or small body, travelling through space and entering the Earth's atmosphere. The same is true for the search of extraterrestrial life on other planets like e.g. on Mars. For these missions it is essential to know whether the detected biomarkers definitely originate from the analysed site or if they could be potential contaminations from “stowaways” which travelled as hitchhikers on the spacecraft or analytical equipment. This potential contamination of space vehicles and subsequently of other planets as well as the outer space has to be assessed and analysed in detail to unambiguously identify extraterrestrial signs of life on the planets and small bodies of our solar system to be able to distinguish them from life on Earth. Furthermore it is a critical point for the field of planetary protection: On the one hand it is important to avoid cross contamination of planets and celestial bodies during human or human caused exploration (forward contamination) and on the other hand to strictly control and analyze extraterrestrial return samples (backward contamination) to detect life forms potentially harmful for our Earth [44]; (Further information on planetary protection is available on NASA's webpage: www.planetaryprotection.nasa.gov).

Therefore, the survival capacity of microorganisms and biomolecules under space conditions as well as after the impact on a planet's surface has to be tested extensively (reviewed in [4], [33], [44]) and a contamination risk evaluation is mandatory to define necessary decontamination strategies (see NASA's webpage: http://solarsystem.nasa.gov/docs/PPCCTECHREPORT3. pdf)

This requires extensive testings of known biomarkers for their stability and survivability in space, during atmospheric passaging on platforms that are easily accessible and can be used with a high frequency for a high throughput of analyses. A wide variety of extremophilic microorganisms as well as biomarkers like nucleic acids and their capability to withstand the hostile conditions of travelling through space as well as leaving and entering a planet's atmosphere, respectively need to be tested. Sounding rockets represent a suitable facility for high frequency testings in contrast to Foton capsule or ISS experiments where the experiment selection phase is very long and the number of experiments is extremely limited due to the limited access to experimental space, the number of experimental uploads and crew time.

In our experiment we showed that sounding rockets, especially the surface of the payload, represent an excellent platform to investigate on a high fequency base the influence of space as well as atmospheric entry conditions on biomarkers like nucleic acids. We examined the stability and integrity of purified plasmid DNA during a ballistic rocket flight and if it retains its biological activity. During the TEXUS-49 mission we attached artificial plasmid DNA carrying a fluorescent marker (enhanced green fluorescent protein: EGFP) and an antibiotic resistance cassette (kanamycin) directly on the outer surface of the payload. Different positions were chosen: 1) directly on the outer surface, 2) in the grooves of screw heads of one of the experimental module and 3) on the bottom side of the payload. Directly after retrieval and back transport of the payload, DNA samples were recovered. Subsequent analyses showed that a fraction of the DNA was still intact and retained its full biological function. We were able to demonstrate experimentally that plasmid DNA attached to the outer surface of the payload of a sounding rocket can withstand a period of residence in space and the re-entry conditions into the Earth atmosphere as well as the landing, and stays intact and active in its function as carrier of genetic information. With this experimental design we present the establishment of a robust and universal functionality assay to test for the stability of DNA, being a model for nucleic acids that could serve as biomarkers in the search for past or present extraterrestrial life.

## Materials and Methods

### Model DNA for atmospheric re-entry-experiment

The expression vector pEGFP-C3 (BD Biosciences Clontech; Fig. S1) was chosen. This plasmid vector consists of 4727 bp and contains a kanamycin/neomycin resistance gene and an enhanced green fluorescent protein gene for bacterial and eukaryotic expression respectively. The plasmid DNA was transformed in *E.coli* K12 for proliferation and after 16 h incubation in LB medium with Kanamycin at a final concentration of 50 µg/ml at 37°C with shaking the plasmid DNA was purified according to the manufacturer's protocol using the Qiagen Maxi-preparation kit (Qiagen: http://www.qiagen.com/products/catalog/sample-technologies/dna-sample-technologies/plasmid-dna/qiagen-plasmid-maxi-kit#resources).

Briefly: The cells were harvested by centrifugation at 6000 g for 15 min at 4°C. 10 ml of buffer P1 is added followed by 10 ml of buffer P2 and 10 ml of chilled buffer P3 to lyse the cells. After an incubation of 20 min on ice, the cell solution is centrifuged for 30 min at 20.000 g at 4°C and applied to the anion-exchange-based Qiagen-tips. The Qiagen-tip is washed twice and the DNA was eluted in 10 mM Tris buffer, pH 8.5 and quality and quantity were checked spectrophotometrically and stored at −20°C.

### Attachment of plasmid DNA to the surface of the TEXUS-49 payload stage

All locations were thoroughly cleaned with 70% ethanol before applying the DNA. 50–100 µg of plasmid DNA in 10 mM Tris buffer, pH 8.5 was applied to 15 different locations on the surface structure of the payload. Samples 1 to 4 were applied directly on the surface, samples 5 to 12 were applied into the grooves of the heads of screws located on one module of the payload, and samples 13 to 15 were applied on the bottom side. After the application, the DNA was dried in a hot air stream and foil was taped over the sample locations in order to protect the DNA from abrasion. Directly before the launch the foil was removed.

Additionally, a positive control (pc1) was applied on the payload surface, dried in a hot air stream and immediately re-dissolved and recovered (after 1 min) and stored at −20°C. A second positive control (pc2) was applied to a spare part of a previously flown payload structure made of the same material, air dried and incubated for the same time span (9 days) as the plasmid DNA on the payload structure during spaceflight.

### Contamination controls

Negative controls were taken by applying 50 µl of Tris buffer on the payload structure (nc1) and into the screw heads (nc2) as well as on the spare part of payload structure (nc3) used for the application of the positive control.

### TEXUS-49 mission profile

**Figure 1:**
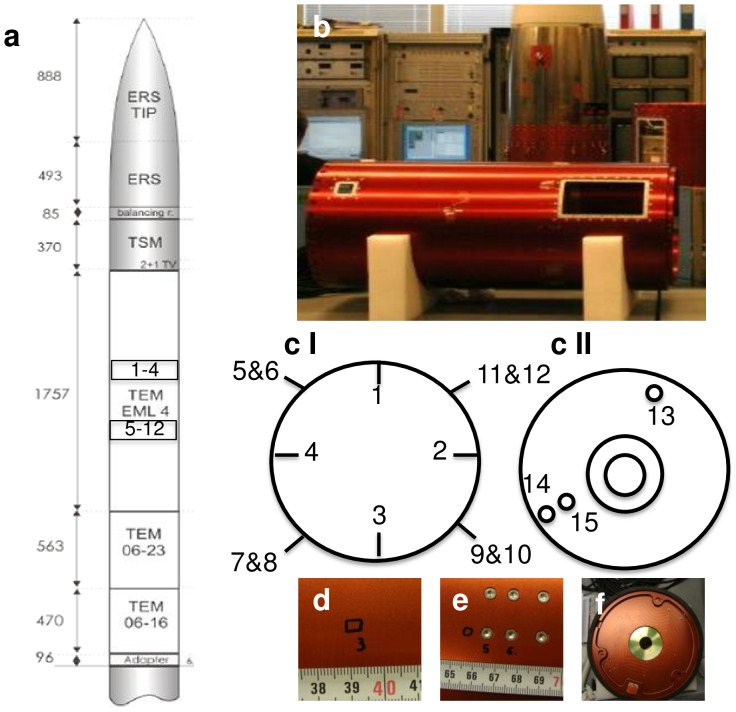
Locations of DNA application on the TEXUS-49 payload. **a** Scheme of the TEXUS-49 payload with DNA sample 1–12 application sites **b** Plasmid DNA samples 1–12 were applied on the outside of the TEM (TEXUS Experiment Module) EML 4 **.cI** DNA samples 1–4 were applied circular at 0, 90, 180, 270 degree directly on the surface of the payload. DNA samples 5–12 were also applied with a distance of 90 degree each in the screw heads of the payload **.cII** DNA samples 13–15 were applied directly on the payload surface at the bottom side **.d** DNA samples 1–4 were pipetted directly on the surface and locations were marked with a pen **.e** DNA samples 5–12 were applied in the grooves of the screw heads **.f** DNA samples 13–15 were applied directly on the payload surface on the bottom side and locations were marked with a pen.

TEXUS-49 was launched nine days after the plasmid DNA was applied to the payload structure. TEXUS-49 consisted of a VSB-30 motor (S-30 solid rocket stage engine with a S-31 second stage engine) and of the payload and was launched on March 29^th^, 2011 at 06:01 a.m., from the ESRANGE (European Space and Sounding Rocket Range) Space Centre near Kiruna, Sweden, north of the Arctic circle (Fig. 1b). During the ballistic suborbital flight an altitude of 268 km and 378 s of microgravity were achieved. Further parameters were: First stage: Peak thrust acceleration 6.3 g, mean thrust acceleration 5.03 g, burnout 12.3 s, motor separation 13.6 s, second stage: Peak thrust acceleration 13.5 g, mean thrust acceleration 7.30 g, burnout 43.0 s, YoYo despin 56.0 s, motor separation 59.0 s. Directly after landing and payload recovery, the DNA was re-dissolved in 50 µl of 10 mM Tris buffer pre-warmed to 60°C and subsequently stored at −20°C.

### DNA quantity and quality measurements of recovered samples

The plasmid DNA quantity and quality of recovered samples were measured spectrophotometrically (Nanodrop 1000, Thermo Scientific) and the DNA recovery rate was determined. 250 ng of each DNA sample were loaded on a 1% agarose gel and the integrity of the DNA was evaluated after electrophoresis by analyzing the degree of DNA degradation.

### Functional test of recovered DNA

The integrity of the plasmid DNA was tested by transformation in *E.coli* and antibiotic selection with kanamycin (50 µg/ml working concentration). 10 ng of DNA was transformed into competent *E.coli* KRX cells according to the manufacturer's protocol (Promega). After incubation in SOC Medium for 60 min at 37°C with shaking, a dilution series was plated on kanamycin (50 µg/ml working concentration) containing agar plates and incubated overnight at 37°C. Colonies were counted and the transformation efficiency was calculated in colonies/ng transformed DNA. For samples with high numbers of bacterial colonies the transformation procedure was repeated with 1 ng of plasmid DNA. Furthermore, a functionality test in NIH-3T3 mouse fibroblasts was performed. Fibroblasts were seeded on glass coverslips and grown in DMEM (4.5 g/L glucose, L-glutamine, and sodium pyruvate; Gibco) containing 10% FCS at 37°C with 5% CO_2_. At 60–70% confluence the cells were washed three times with Opti-MEM (Gibco) and transfected with 1.6 µg of plasmid DNA or 5 µl of the negative control nc1 using Lipofectamine 2000 (Invitrogen) according to the manufacturer's protocol. Cells were incubated for another 24 h at 37°C with 5% CO_2_ before pictures were recorded with a Nikon TS1000 Eclipse microscope and a GFP filter.

### Sequencing of recovered DNA after transformation in *E.coli*

Nineteen different primers were designed to fully cover the pEGFP-C3 sequence and 10 clones from transformation reactions in *E.coli* KRX of samples 1 and 14 each were chosen, inoculated in LB medium and incubated over night at 37°C with shaking. Plasmid DNA was isolated (Qiagen DNA Minikit) and sent for sequencing (SEQLAB). The sequences were edited, aligned with the CAP3 sequence assembly program and compared to the sequence of the original pEGFP-C3 plasmid with Blast 2 Sequences (http://blast.ncbi.nlm.nih.gov/Blast.cgi?PAGE_TYPE=BlastSearch&PROG_DEF=blastn&BLAST_PROG_DEF=megaBlast&BLAST_SPEC=blast2seq).

### Scanning electron microscopy

Pieces of the metal payload structure with a size of 1×1 cm were cut out and the cutting edge was polished with sandpaper of different grain size (200, 400, 600, 800, 1000 µm). The payload structure pieces were cleaned in distilled water containing detergents and sonicated in an ultrasonic bath 3 times for 5 min. Each metal piece was rinsed at least 3 times in distilled water and subsequently 3 times in 96% ethanol and 3 times in aceton. 50 µg of pEGFP-C3 plasmid DNA or 50 µl of 10 mM Tris buffer were applied and the solutions were dried in a hot air stream. The samples were mounted on the sample holder with carbon adhesive Leit Tabs G3347 (Plano GmbH, Germany), inserted into the sample chamber and observed with the Hitachi S450 scanning electron microscope.

### Numerical and statistical analysis

For DNA recovery and DNA integrity rates average values and standard deviations were calculated for all analyzed groups. The results were compared statistically using a two tailed unpaired Student's t-test. Values were analyzed in pairwise combinations and values ≤0.05 were considered as statistically significant. Statistical significance is represented by asterisks corresponding to *P<0.05, **P<0.01, ***P<0.001.

## Results

### Exposure of DNA to launch, space flight and re-entry into Earth's atmosphere

DNA of the plasmid expression vector pEGFP-C3 (Fig. 1f) was applied to the external surface of the payload stage mounted on a VSB-30 two-stage solid-fuel rocket (Fig. 1a–e), which was launched during the TEXUS-49 campaign on March 29^th^, 2011 from the ESRANGE Space Center in Kiruna, Sweden. DNA samples were applied to fifteen different locations: 1.) directly on the surface, 2.) into grooves of the head of screws located on the outside of the payload, and 3.) on the bottom side of the payload structure (see material and methods; Fig. 1). Simultaneously, two positive controls (pc) were applied, pc1 on the payload structure, which was immediately recovered and pc2 on a previously flown payload structure, which was recovered after nine days together with samples 1–15 after the flight in order to test for the impact of desiccation on the DNA integrity.

**Figure 2:**
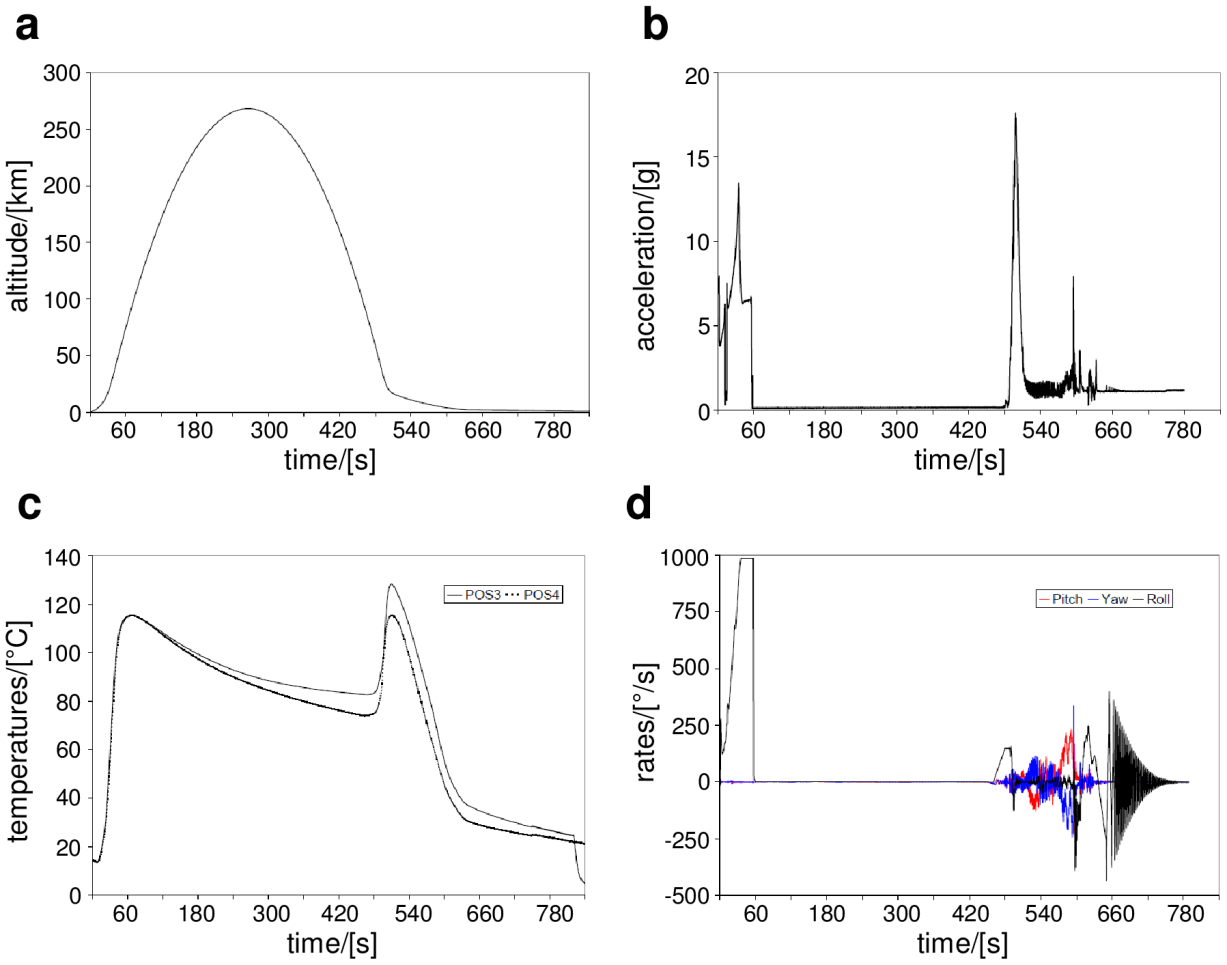
TEXUS-49 telemetry flight data. **a** altitude, **b** acceleration, **c** temperature and **d** pitch, roll and yaw angles of rotation.

The TEXUS-49 flight lasted 780 seconds, including 378 seconds of microgravity with a quality of 10^−5^ g. The payload reached an altitude of 268 km (Fig. 2a) and experienced two hypergravity phases, one during the start with a peak acceleration of 13.5 g and a second one during the atmospheric re-entry with 17.6 g (Fig. 2b). The temperature was monitored with two independent sensors on the inside of the payload wall and reached a maximum of 115.4°C during the start phase and 128.3°C during the atmospheric re-entry (Fig. 2c). The monitoring of the rotation in the three dimensions pitch, roll and yaw confirm the atmospheric re-entry phase at approximately 480 seconds (Fig. 2d).

### DNA recovery after landing

**Figure 3:**
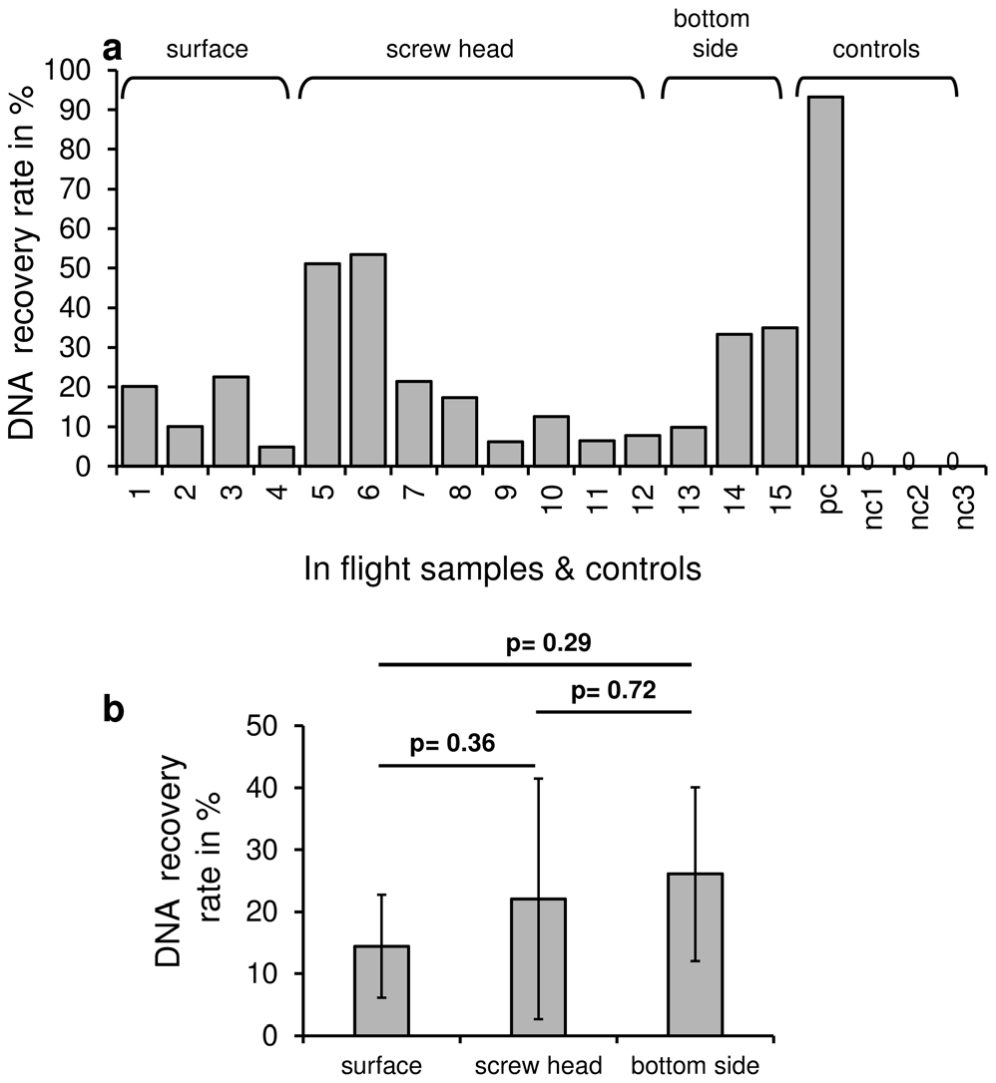
DNA recovery rate after re-entry and landing. After post-flight payload retrieval, DNA samples were recovered and dissolved in Tris buffer. The DNA concentration was measured and the recovery rate was calculated with respect to the initial application concentration. DNA was detected in all the analyzed samples pc = positive control; nc = negative control **a** DNA recovery rate at single sample positions, **b** average DNA recovery rate for surface (n = 4), screw head (n = 8) and bottom side (n = 3) (SD, two-tailed unpaired t-test; p-values are shown for respective pairwise analyses).

After the landing site of the payload was tracked by GPS and the payload was returned to the laboratory, the DNA was recovered by re-dissolving it in Tris buffer previously warmed to 60°C. The DNA concentration and purity was analyzed spectrophotometrically. Potential protein contamination was investigated by calculation of the ratio of absorptions at 260 nm versus 280 nm. Twelve of the fifteen samples showed a high degree of purity with 260 nm/280 nm absorption ratios of 1.71–1.85. For 3 samples ratios between 1.56–1.68 were measured indicating a low level of protein contamination, which most likely occurred during the process of landing. Based on the measured DNA concentration after sample recovery the DNA recovery rate was calculated in relation to the amount of DNA that had been applied (Fig. 3). In total, 15 samples, two positive controls and three negative controls were analyzed. In all 15 samples and the two positive controls DNA was detectable (Fig. 3a). The recovery efficiency ranged between 4.9% and 53.4% for the samples and 39.3% and 93.2% for the positive controls, respectively. In the three negative controls no DNA was detectable indicating that there was no considerable contamination present on the sounding rocket payload structure. The comparison of the DNA recovery at the different application sites shows that on average the highest amount of DNA was recovered on the bottom side of the payload (Fig. 3b), indicating that this is the location with the highest level of protection during the atmospheric entry.

### Assaying DNA integrity

**Figure 4:**
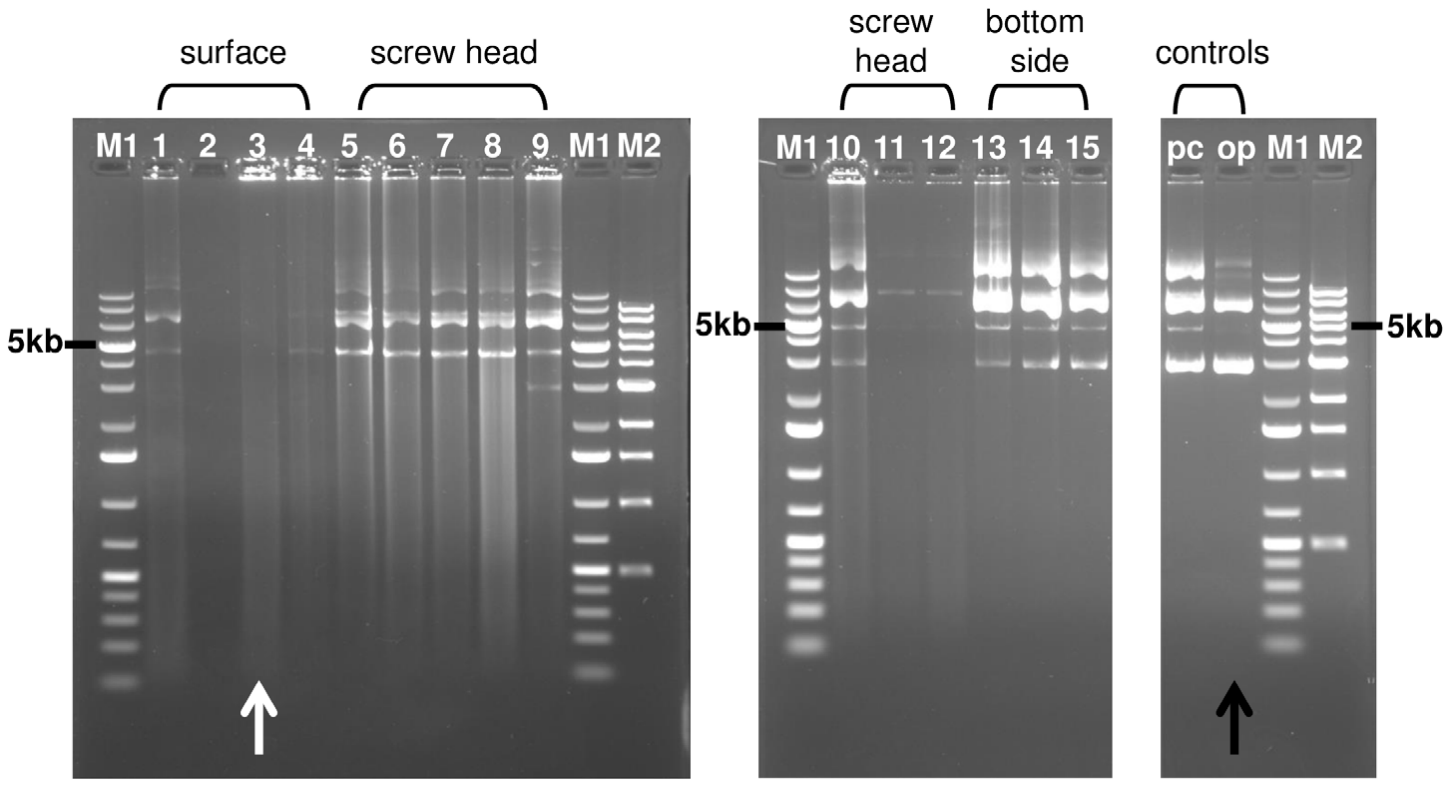
DNA quality analysis by agarose gel electrophoresis. For each sample 250 ng of DNA was loaded on an agarose gel and the degree of degradation was analyzed after the run. The highest degree of degradation was recorded for samples 1–4, which were applied directly to the surface of the payload. For the samples applied in the grooves of the screw heads a lower degree of degradation was observed. DNA with the highest integrity was observed for the samples applied on the bottom side of the payload. The white arrow depicts the sample with the highest degree of degradation. The black arrow points to the lane where the original plasmid (op) was loaded. M1: Marker Gene Ruler 1 kb ladder; M2: Marker 1 kb DNA ladder; pc: positive control (9 days ground control).

The degree of DNA degradation caused by the conditions during the flight was further investigated by loading the DNA samples on agarose gels. DNA integrity was estimated by the degree of visible DNA degradation. In addition to the positive controls the same amount of the original plasmid was loaded on the gel to compare DNA fragment sizes and intensities as well as the degree of degradation represented by the smear appearing below a size of 5 kb (Fig. 4). Only two prominent fragments were visible in the lane of the original plasmid, in samples 7, 8, 9, 10, 13, 14, 15 and the positive controls, an additional fragment of approximately 10 kb appeared. This could be a desiccation effect, leading to the formation of more complex non-reversible structures. Furthermore, an additional band appeared at the height of approximately 5 kb which represents nicked, linearised plasmid DNA. In samples 1, 3, 4, 5, 6, 7, 8, 9, 10, 11, 12 a smear from 5 kb down to 250 bp became visible, indicating a high degree of degradation caused by the in-flight conditions. In sample 2 the degree of DNA degradation was so prominent, that no signal was detectable in the agarose gel, despite the results of the spectrophotometrical investigation. The agarose gel analysis confirmed that DNA applied to the bottom side of the payload had the highest degree of integrity followed by the samples applied in the grooves of the screw heads (Fig. 4).

### Functional activity in *E. coli*

**Figure 5:**
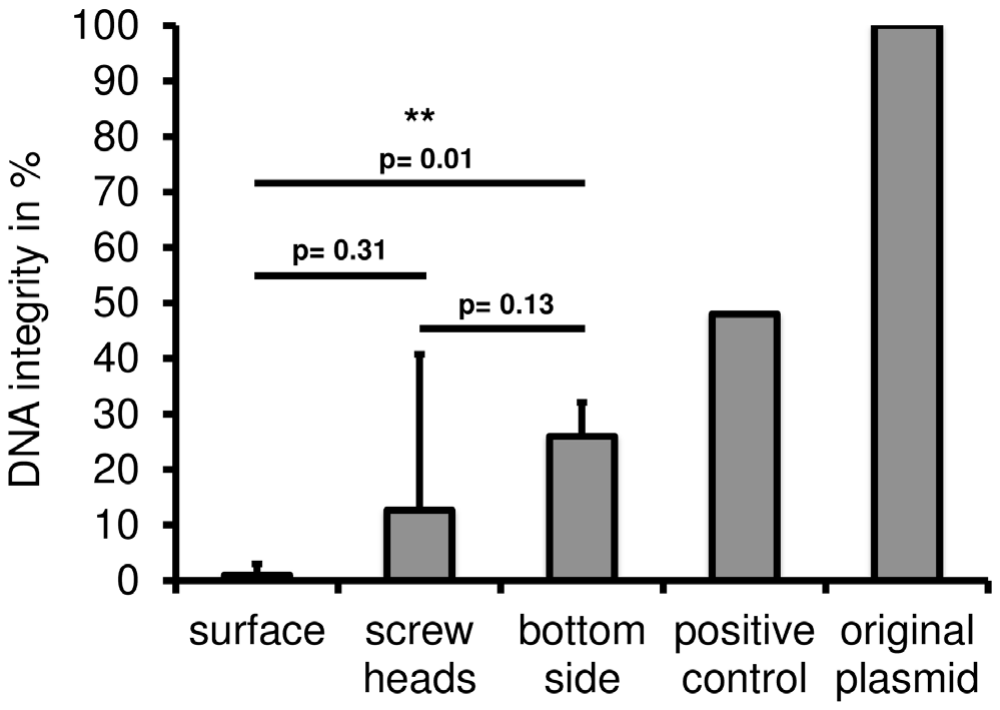
DNA integrity after transformation. Recovered plasmid DNA was transformed in *E coli* KRX and the integrity was calculated based on the transformation efficiency in colonies per nanogramm DNA. A significant increase in the degree of DNA integrity was detected for the samples applied on the bottom side of the payload compared to the surface samples (p-value = 0.01). (surface samples: n = 4; screw heads samples: n = 8; bottom side samples: n = 3; positive control: n = 1, original sample: n = 1) (SD, two-tailed unpaired t-test; p-values are shown for respective pairwise analyses).

After the measurement of the quantity and quality of the recovered DNA, we tested the functional activity of the plasmid DNA. pEGFP-C3 carries a kanamycin resistance gene which provides antibiotic resistance to bacteria after transformation. Therefore, pEGFP-C3 plasmid DNA was transformed in *E. coli* KRX and plated on kanamycin agar plates to determine the amount of intact plasmid DNA per sample. The transformation efficiency was calculated in colonies per nanogramm of transformed DNA (Table 1). The transformation efficiency for the original plasmid DNA is 1955 colonies/ng transformed DNA and was set to 100%. For DNA samples 2 to 4, applied directly to the payload surface, no bacterial colonies were identified, indicating that the degree of DNA damage was so high that only a minority of plasmid DNA molecules stayed intact, undetectable with these low amounts of DNA used for the transformation. However, for sample 1, 69 colonies/ng DNA were identified, showing that also on the surface of the payload with the lowest degree of protection, intact DNA molecules were present. For all samples applied in the grooves of the screw heads, bacterial colonies were detected. Despite the low number of bacterial colonies counted for samples 5, 6, 7, 8, 11, 12, for sample 8 the transformation efficiency was 138 colonies/ng DNA and for sample 10 even 1368 colonies/ng DNA. These findings demonstrated that the grooves of the screw heads sufficiently protected the applied DNA from damage. Samples applied to the bottom side of the payload had the highest integrity with a transformation efficiency of 432 and 644 colonies/ng DNA. For the positive control, we obtained 932 colonies/ng DNA indicating that 48% of the DNA was still intact after desiccation and incubation at room temperature for the duration of the mission (9 days). Negative controls were simultaneously tested and no bacterial colonies were detected on these agars plates indicating that the experimental conditions were sufficiently clean to not produce false positive signals (Table 1, Fig. 5).

**Table 1:** Functional activity of recovered plasmid DNA in bacteria.

Sample no	Location	Amount of transformed DNA	Total number of bacterial colonies	Transformation efficience colonies/1 ng DNA	DNA integrity [%]
1	Surface	10 ng	690	69	4
2	Surface	10 ng	0	0	0
3	Surface	1 ng	0	0	0
4	Surface	1 ng	0	0	0
5	Screw head	1 ng	69	69	4
6	Screw head	10 ng	6	∼1	0,05
7	Screw head	10 ng	45	∼5	0,3
8	Screw head	10 ng	43	∼4	0,2
9	Screw head	1 ng	138	138	7
10	Screw head	1 ng	1368	1368	70
11	Screw head	10 ng	170	17	0,9
12	Screw head	10 ng	244	24	1,2
13	Bottom side	10 ng	4324	432	22
14	Bottom side	10 ng	4508	451	23
15	Bottom side	1 ng	644	644	33
pc2	Ground module	1 ng	932	932	48
nc1	Flight module	5 µl	0	0	–
nc2	Flight module	5 µl	0	0	–
nc3	Flight module	5 µl	0	0	–
pEGFP-C3	Original plasmid DNA	1 ng	1955	1955	100

### Functional activity in mouse fibroblasts

**Figure 6:**
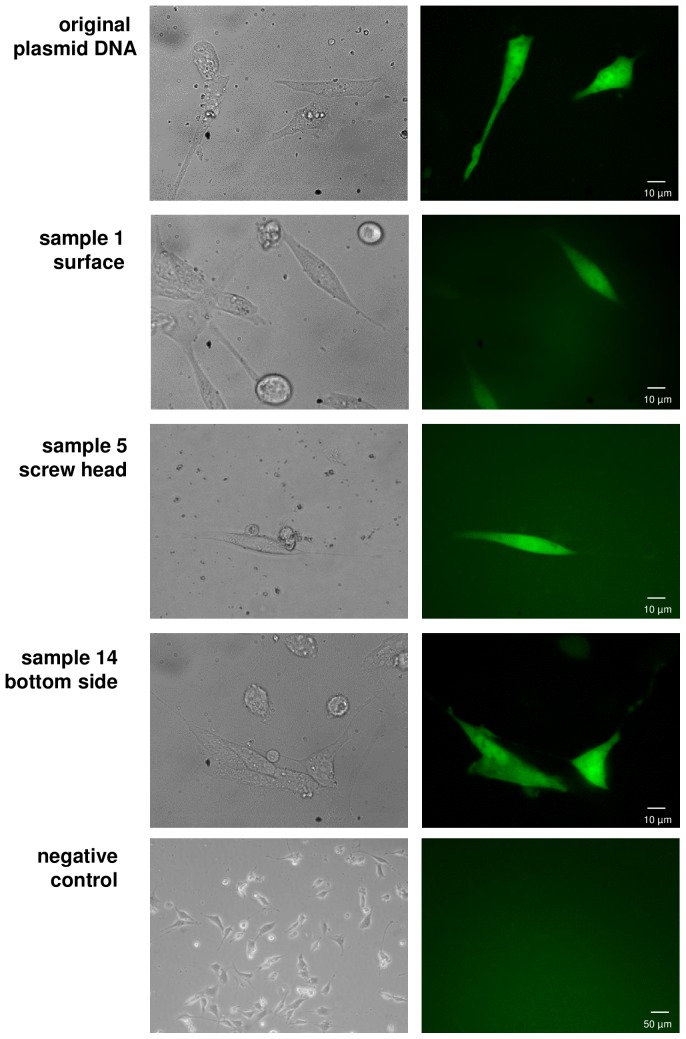
DNA function in eukaryotic cells. For each application location (surface, screw head, bottom side) one recovered DNA sample was chosen for transfection into NIH-3T3 mouse fibroblasts and EGFP expression was visualized after 1 day of incubation. In all the samples EGFP expression was detectable indicating the functionality of the DNA samples. Left column: Phase contrast images; Right column: GFP filter.

A second functionality test was performed by transfection of plasmid DNA applied on the surface, in the grooves of the screw heads and the bottom side of the payload into NIH-3T3 mouse fibroblasts. Samples 1, 5, and 14 were chosen to represent the three different application sites and nc1 was used as a negative control. One day after transfection, the samples were analyzed microscopically for the expression of the GFP-marker located on the plasmid. For all transfected DNA samples GFP expression with similar intensities was detected (Fig. 6). The negative control nc1 showed no GFP signal and no autofluorescence could be detected in the fibroblasts. This second functionality test in eukaryotic cells also showed that intact circular plasmid DNA remained post-flight enabling the expression of the GFP marker.

### Flight induced mutagenesis analysis

The next step was to examine whether the DNA sequence was 100% homologous to the original plasmid DNA or if point mutations were generated due to the in-flight conditions. DNA of 10 bacterial colonies from the transformations of sample 1 and sample 14 was isolated and sequenced with nineteen different primers to obtain a 2 to 4 fold sequence coverage. The resulting 320 sequences were assembled and contigs were generated to perform a Blast-2-sequences analysis against the original plasmid DNA sequence data. All colonies of sample 14 and 9 colonies of sample 1 showed a 100% homology to the original sequence. In one colony of sample 1 two point mutations were identified, one insertion of a cytosine and one deletion of a thymine preventing a frame shift and allowing the correct expression of the antibiotic resistance and the GFP-marker. In total, only 0.001% of the analyzed sequence information of sample 1 contained mutations induced by the flight conditions (Table 2).

**Table 2:** Flight induced mutagenesis anlysis by sequencing.

sample #	application site	# colonies sequenced	# analysed sequences	# colonies with mutation	# of point mutations	% flight induced mutagenesis
1	surface	10	160	1	2	0 001
14	bottom side	10	160	0	0	0

### Surface structure and DNA localization

**Figure 7:**
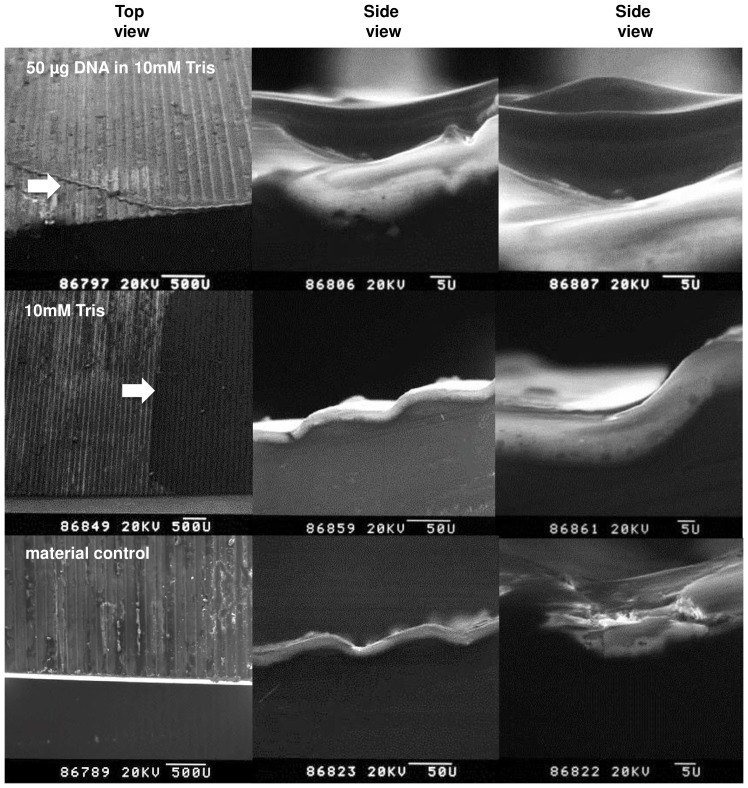
Scanning electron microscopy (SEM). The surface of the TEXUS payload was analyzed by SEM 50 µg of DNA in Tris buffer was applied and air dried. As controls the same volume of Tris buffer was applied and air dried and additionally the structure was analyzed without an application of fluid. The DNA Tris buffer mixture forms a thick film that completely fills the grooves with a width and a height of approximately 100 µm and 21 µm, respectively. Arrows indicate the location where DNA or Tris buffer solution was applied.

Finally, we investigated the surface of the payload structure in more detail and we examined how the DNA is arranged and distributed. Small pieces of 1×1 cm of the payload structure were cut out and 50 µg of the original plasmid DNA was applied and dried in a hot air stream. The samples were analyzed by scanning electron microscopy. The high resolution of this technique allows to visually measuring the size of the grooves on the surface of the material formed by the manufacturing process. The grooves have a width of about 100 µm and a height of approximately 21 µm. The pictures taken without DNA and buffer and with Tris buffer only show that the material is covered with an eloxed layer (Fig. 7). The application of 50 µg plasmid DNA in Tris buffer and the following desiccation resulted in DNA in crystallized form that completely filled up the grooves of the material. To a certain degree this relatively thick layer of 21 µm of crystallized DNA material probably served as a protective shield so that numerous DNA molecules were kept functionally intact to enable antibiotic resistance in bacterial cells and GFP-marker expression in eukaryotic cells after transformation and transfection respectively.

## Discussion

In the search for extraterrestrial signs of life, space technology plays an important role and offers many different platforms to explore the planets and small bodies in our solar system, including sample collection and on-site analysis as well as to use the specific platform conditions to investigate the effect of the space environment on biomolecules and whole organisms.

### Analysis of DNA stability and integrity

Here we described the utilization of a robust and univeral experimental setup to evaluate the stability of plasmid DNA, acting as biomarker model, towards space conditions. Agarose gel electrophoresis and transformation and transfection techniques into bacterial and eukaryotic cells were performed to test for the DNA integrity as well as for the remaining biological function. We could show that purified plasmid DNA applied to the surface and in screw heads, circular all around the payload, as well as to the bottom of the payload of a sounding rocket can withstand the hostile conditions prevailing during the atmospheric passages on a ballistic flight (recovery of DNA in all 15 samples with a recovery efficiency between 4.9% and 53.4%). DNA samples were exposed for approximately 400 sec to space above 100 km and in total for about 200 sec to temperatures above 100°C. It can be assumed that among temperature, radiation, pressure, desiccation, and microgravity, temperature has the highest impact on DNA integrity during the 13 min of total flight time during the TEXUS-49 mission. Despite the hostile conditions during atmospheric passage, DNA recovery was possible from all sites of application: 1.) payload surface, 2. screw heads, and 3.) bottom side of the payload but to different extents. Furthermore, the two functionality tests in prokaryotes and eukaryotes revealed that DNA samples were still biologically active. In bacterial transformation assays, transformation efficiency ranges between no or only a few bacterial colonies (69 colonies/ng DNA recovered from payload surface with the lowest degree of protection) until 1368 colonies/ng DNA (recovered from grooves of the screw heads). In eurkaryotic transfection assays, all recovered DNA samples which demonstrated capability of bacterial transformation, were also capable of transfecting NIH-3T3 mouse fibroblasts to express GFP. The data show that the integrity of the DNA increased in the following order: payload surface>screw head>bottom side, according to the level of exposure to high temperatures. Directly at the surface on the side of the payload, conditions are harshest and high structure temperatures of 130°C and gas temperatures up to 1000°C are likely. Therefore, DNA showed the highest degree of degeneration at these sample application sites (Fig. 4).

### Protection of DNA against degradation

Due to the spin of the payload during ascent and the circular application of the samples, all application sites were equally exposed to high temperatures of up to 118°C. Additionally, the DNA was exposed to temperatures between 80–100°C during the microgravity phase and up to 130°C during ascent and descent phase. The random exposure of the samples to the constantly high temperatures weakens the phosphodiester bond which leads to hydrolysis resuling in single and double strand breaks [37]–[38]. The temperature induced DNA damage could explain the different degrees of degradation visible in the agarose gel electrophoration. Futhermore, it is likely that sample quantities are partially reduced during the landing procedure when the payload is landing on ice and snow. Compared to the sample application directly onto the payload surface, small niches in the grooves of the screw heads provided a certain degree of protection against maximum temperatures, and we observed the highest degree of protection on the bottom side. However, intact DNA was detectable for all sample application sites. We therefore conclude that the minimal protection given by the grooves on the surface of the payload structure, as well as by the grooves of the screw heads combined with the observed relatively thick layer (21 µm) of DNA and salt crystals was sufficient for at least a fraction of the DNA molecules to stay intact and retain their full function. The protective role of so called biofilms have been already shown for spores (reviewed in [26]). Additionally, high salt concentration can have a beneficial effect on DNA denaturation and degradation. Salts like MgCl_2_ and KCl counteract the denaturation of double-stranded DNA at high temperature, and renaturation of single-stranded products of thermodegradation is favoured when the temperature of the incubation mixture decreases. A protective effect of salt crystals for the survival of bacteria under vacuum and UV conditions were reported also earlier by [45]. The fact that DNA is suceptible to high temperature was already shown by several groups before [46]–[48]. Chiter and colleagues [49] who analysed the transfer of genetic modified organisms in form of DNA fragments could show that temperatures above 95°C for more than five minutes lead to a high level of DNA fragmentation so that it is unlikely that genetic information is retained.

However in our experiment we could show that in contrast to DNA fragments, the application of small circular DNA designed on a bacterial plasmid background, on the outer side of the structure of the payload of a sounding rocket can withstand at least partially even higher temperatures of up to 130°C. The choice of location of application is crucial for the integrity of the biomolecule and is highest for locations at the bottom side of the payload. Considering the aspect of a biomolecular based search approach for the detection of potential life outside our Earth a detailed analysis of the biomarker or micoorganism stability as well an investigation of the vehicle with respect to microorganism and biomolecular contaminations should be taken into account as well.

### Biomarker stability and survivability assay on different platforms

The experimental setup that is described here represents an analysis method to determine the survival rate of the biomarker of interest and suggests the locations with the highest risk of integrity of biomarker where an intense decontamination is needed. Therefore, the described experimental setup represents a new valuable combination of tools to characterize the stability of nucleic acids being an important biomarker in the search for extraterrestrial past or present life.

Besides nucleic acids as a biomarker, the survival of microorganism and their spores is of high interest. A number of experiment series have already been performed during the STONE 1–6 missions on the Russian Foton satellite to address the question of microbial survival. Physical, chemical, and biological modifications caused by atmospheric entry in rocks, meteoritic materials, and embedded microorganisms were investigated [50]. The aim was to analyze if the rock material prevailing on the surface of Mars could withstand the entry into Earth's atmosphere and if simple life forms embedded in these rocks could survive these hostile conditions. Different types of rocks, loaded with or without microorganisms, were fixed to the heat shield of the Foton return capsule, exposed to space conditions for two weeks and subjected to atmospheric re-entry conditions similar to those of meteorites [50]. From the first four series of experiments (STONE 1–4) material returned only from the Stone 1 mission and it was shown that temperatures during re-entry were high enough to form a melting crust on the rocks [50]–[51]. The STONE 1 experiment was repeated during the STONE 5 experiment where besides different stone materials [28], [52] also the survival of different microorganisms like endolithic cyanobacteria, bacterial and fungal spores, and dried vegetative cryptoendoliths on and inside the rocks were analyzed for their survival of atmospheric entry [28]. Post-flight analyses revealed that all rock samples showed a melting crust on their surface. Temperatures were measured to reach a maximum of 1840°C at the surface, indicating that this experiment, despite the lower atmospheric entry speed of 7.6 km/s instead of 20–70 km/s, simulated the atmospheric entry of a meteorite [52]. After sample recovery, the analyses showed that the microorganisms did not survive the atmospheric re-entry. The rock layer of 5 mm was not thick enough to protect against the high temperatures [28]. The same Foton satellite also hosted the Biopan 5 container carrying lichens. These microorganisms were exposed to space conditions for 16 days, but for the atmospheric entry the container was closed to shield the samples from high temperatures and pressure. The analysis of the microorganisms showed that some of them survived the space conditions and 24 hours after return to Earth their metabolism normalized completely [17]. In a follow up experiment (STONE 6) microorganisms even protected by a 2 cm thick layer of rock did not survive [30]. Calculations showed that a rock needs to be at least 5 cm thick [30] in order to not exceed temperatures of 121°C inside the material, the maximal temperature for hyperthermophilic life [53]. The STONE experiment series show that the Foton capsule is a highly valuable platform to simulate atmospheric entry but the experimental reproducability is extremely limited due to the limited launch events and can not fully serve the need requested by the researchers for the high frequency of follow-up experiments.

### Sounding Rockets as experimental testbed

Despite the negative results concerning microorganism survival during the STONE experiments, Fajardo-Cavazos and colleagues used the ballistic flight of a sounding rocket to test the survival and degree of induced mutagenesis of *Bacillus subtilis* WN511 spores during the ascending and descending atmospheric passage phases [27]. A granite samples was soaked with bacterial spores and divided into five sections: top, left, right, front, and back, with “front” being the leading edge of the sample during flight [27]. Spore survivors were found for all sections (1.2% to 4.4% in comparison to ground controls) but not at the front section indicating that at this sampling site the atmospheric passage conditions were too harsh and completely sterilized the material. However, at the other sampling sites the spores were able to survive the flight. Sporulation-defective mutants were identified with a high frequency of 4%. The exclusion of possible environmental contaminants was confirmed by molecular fingerprinting using RAPD-PCR and rep-PCR [27].

In contrast to our experiment, here only one sampling site was tested and devided into 5 sampling groups meaning that only a small area was analyzed. Due to the limitation of only one sample application site, there was no overall survey of conditions prevailing during the two atmospheric passages on the complete outer side of the sounding rocket. The spin of the payload especially during ascent would require a circular sample application around the complete payload structure to mirror an exact picture of the influence of the prevailing conditions on the samples. A slight modification of the experiment consisting of an evenly distributed sample application all around the payload structure would most likely give a more detailed overiew of the forces acting during the flight on the investigated samples. With our experimental setup, covering the full radius of the payload, we could show that at least for macromolecules like DNA much less protection is necessary than for microorganisms, since functional DNA was recovered from all application sites. Additionally, the used artificial DNA has the high advantage that it is a biomarker which can be unambiguously identified so that less complex environmental controls are necessary. We recommend using plasmid DNA as a representative test biomarker to analyze the applicability of platforms and scenarios in space research. Furthermore, this form of assay allows a simple and robust direct analysis of DNA quality and quantity by a combination of gel electrophoresis, transformation, transfection and sequencing. In addition, usage of artificially designed plasmid DNA is beneficial, because it is uniquely identifiable and a direct analysis without intermediate amplification steps can be applied.

## Conclusions

In our experiment we could show that sounding rockets are an ideal platform to study the impact of high velocity atmospheric passage combined with short term microgravity, UV, and high temperature exposure. We demonstrated stability and functional activity of DNA during hypervelocity atmospheric transit. This platform-biological-assay combination should be extensively used to investigate the influence of atmospheric passage conditions and short term microgravity on further biomarkers, as well as on different microorganisms, including extremophiles. Including slight modifications of the experiment design concerning the payload structure it will also be possible to study the effects of the single phases of a sounding rocket flight resulting in a more detailed picture of the influence of the single forces like radiation, pressure, microgravity and temperature. The here presented experimental setup delivers a excellent tool to make a big step forward in the characterization of biomarkers and microorganisms and their behaviour that is necessary for the extraterrestrial search for life as well as for the field of planetary protection.

## Supporting Information

Figure S1 Vector map of pEGFP-C3: The expression vector pEGFP-C3 (BD Biosciences Clontech) consists of 4727 bp and contains a kanamycin/neomycin resistance gene and an enhanced green fluorescent protein gene for bacterial and eukaryotic expression respectively.
(TIF)
