# Supplementary figures and images for: Functional Activity of Plasmid DNA after Entry into the Atmosphere of Earth Investigated by a New Biomarker Stability Assay for Ballistic Spaceflight Experiments
Source: PLoS One. 2014 Nov 26;9(11):e112979. doi: 10.1371/journal.pone.0112979 (PMC4245111; doi:10.1371/journal.pone.0112979)

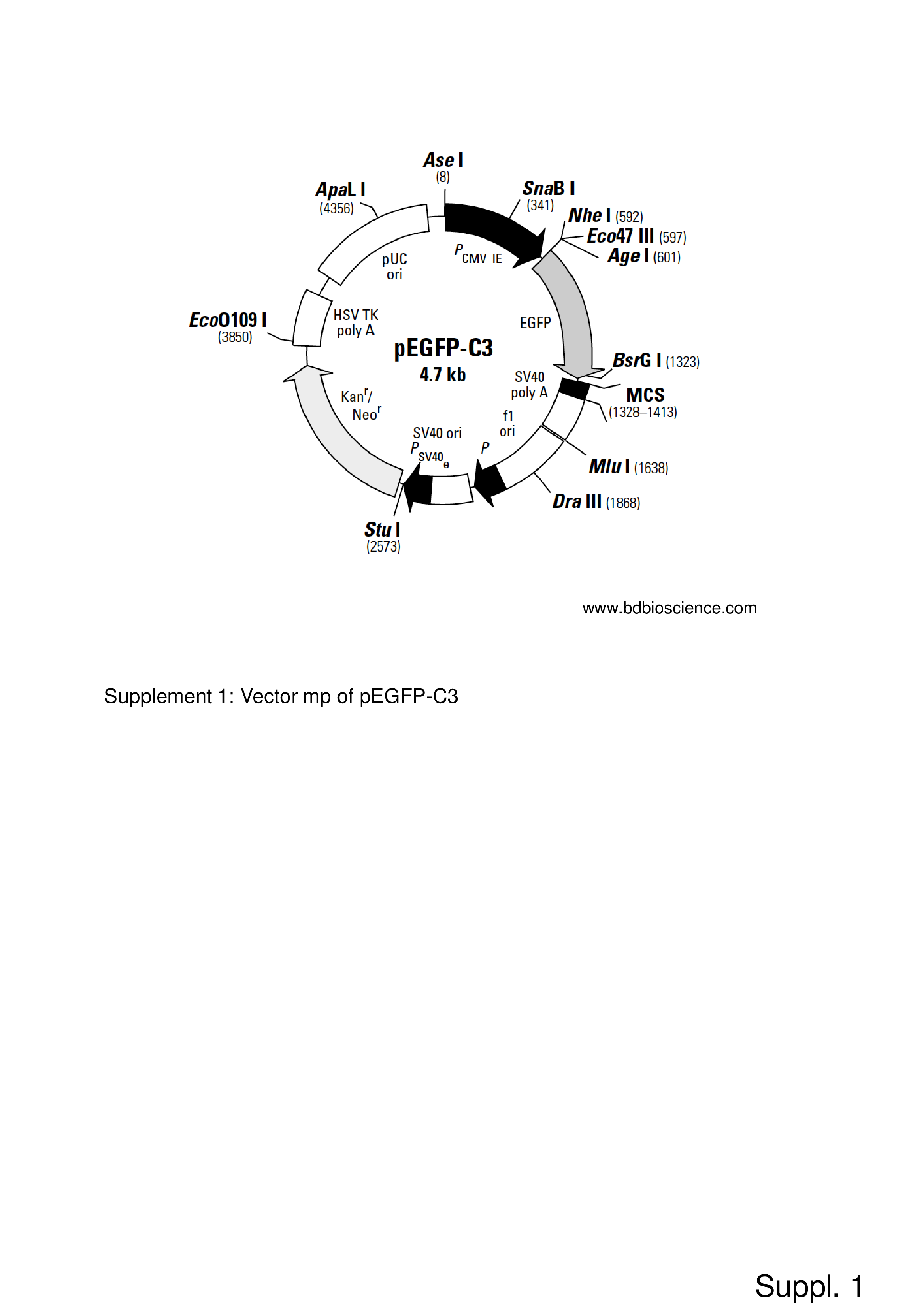

Supplement: Figure S1 — Vector map of pEGFP-C3: The expression vector pEGFP-C3 (BD Biosciences Clontech) consists of 4727 bp and contains a kanamycin/neomycin resistance gene and an enhanced green fluorescent protein gene for bacterial and eukaryotic expression respectively. (TIF) [file pone.0112979.s001.tif]
